# Why Unidimensional Pain Measurement Prevails in the Pediatric Acute Pain Context and What Multidimensional Self-Report Methods Can Offer

**DOI:** 10.3390/children6120132

**Published:** 2019-12-02

**Authors:** Tiina Jaaniste, Melanie Noel, Renee D. Yee, Joseph Bang, Aidan Christopher Tan, G. David Champion

**Affiliations:** 1 Department of Pain and Palliative Care, Sydney Children’s Hospital, Randwick, NSW 2031, Australia; renee.yee@student.unsw.edu.au (R.D.Y.); joseph.bang@student.unsw.edu.au (J.B.); dchamp@bigpond.net.au (G.D.C.); 2 School of Medicine, University of New South Wales, Sydney, NSW 2052, Australia; aidan.tan@unsw.edu.au; 3 Department of Psychology, University of Calgary, Calgary, AB T2N 1N4, Canada; melanie.noel@ucalgary.ca; 4 Alberta Children’s Hospital Research Institute, Calgary, AB T3B 6A8, Canada; 5 Hotchkiss Brain Institute, Calgary, AB T2N 1N4, Canada

**Keywords:** child, pain assessment, multidimensional, affective, evaluative, intensity

## Abstract

Although pain is widely recognized to be a multidimensional experience and defined as such, unidimensional pain measurement focusing on pain intensity prevails in the pediatric acute pain context. Unidimensional assessments fail to provide a comprehensive picture of a child’s pain experience and commonly do little to shape clinical interventions. The current review paper overviews the theoretical and empirical literature supporting the multidimensional nature of pediatric acute pain. Literature reporting concordance data for children’s self-reported sensory, affective and evaluative pain scores in the acute pain context has been reviewed and supports the distinct nature of these dimensions. Multidimensional acute pain measurement holds particular promise for identifying predictive markers of chronicity and may provide the basis for tailoring clinical management. The current paper has described key reasons contributing to the widespread use of unidimensional, rather than multidimensional, acute pediatric pain assessment protocols. Implications for clinical practice, education and future research are considered.

## 1. Introduction

A comprehensive and concise assessment of acute pediatric pain is critical in helping health professionals understand a patient’s pain experience, identify changes in the patient’s condition, informing clinical responses, and establishing the efficacy of a clinical response. Acute pain may be taken to include all painful experiences lasting for less than 3 months duration [1]. A thorough acute pain assessment should be conducted within a broader social and developmental perspective, considering information from various sources, which may include self-reported information, behavioral observations, physiological measures, parent- or clinician-reported information, and the patient’s medical history [2], complemented by an understanding of pain mechanisms and pathophysiology [3]. Each of these assessment modalities may provide valuable information about the pain experience. However, given the subjective nature of pain, eliciting self-reported information is arguably the method most nuanced to provide multidimensional information about the pain experience, albeit not without limitations (for a review of limitations see [4,5]). Moreover, the opportunity to attain quantifiable self-reported responses or scores on various dimensions of the pain experience has the potential to be invaluable not only for research purposes, but clinically for sharing standardized information between health professionals, tracking change, assessing risk for poor pain outcomes, and tailoring therapeutic interventions. The focus of the current review is on the nature and importance of eliciting multidimensional self-reported information about the pediatric acute pain experience.

Although pain is widely recognized to be a multidimensional experience and defined as such, the use of unidimensional pain measures focusing on quantifying pain intensity prevail in the pediatric acute pain context. Experienced clinicians may follow a unidimensional pain intensity assessment with additional ad hoc questioning which may provide some sense of the affective or cognitive pain domains, but this is rarely recorded in emergency department records, ward-based nursing documentation, or on acute pain rounds. Unidimensional assessments that are recorded fail to provide a comprehensive picture of an individual’s qualitative experience. Considerable attention has been devoted to the development of age-appropriate unidimensional, sensory measures of pain [6,7]; increasingly though the clinical utility of unidimensional pain intensity measures has been called into question (e.g., [5]). It has been suggested that describing pain only in terms of intensity is like describing music only in terms of loudness [8]. Moreover, unidimensional pain scores commonly yield little information on which to base clinical decisions [5]. These issues are not unique to the pediatric context, with similar concerns documented in the acute pain context with adults [9,10,11].

While a strong case has been made elsewhere for the value of multidimensional acute pain assessment for the purposes of a diagnostic aid and achieving more nuanced acute pain taxonomy [12], the current paper has adopted a broader conceptual focus on the importance of eliciting multidimensional self-report information about a child’s pain experience, giving consideration to the potential benefits this may confer on clinical response and outcomes. This paper will begin with an overview of the theoretical and empirical literature supporting the necessity of assessing pediatric acute pain from a multidimensional perspective. The literature on the concordance of children’s self-reported sensory and affective pain scores will be considered, specifically in acute pain contexts. Associations between acute pain dimensions and pain chronicity will be considered, as will implications for therapeutic response. This paper will outline key reasons contributing to the widespread use of unidimensional, rather than multidimensional, acute pain assessment protocols. Implications for future research and clinical practice will be discussed.

We performed a narrative literature review, using strategies widely used in systematic reviews, searching the electronic bibliographic databases MEDLINE (via Ovid) and EMBASE (via Ovid) from their inception until October 2019. Given the focus of the paper, we did not perform a systematic review or meta-analysis. We searched for articles which utilized self-report measures of children’s acute pain directed at two or more of the following dimensions of the pain experience: sensory, affective, and cognitive-evaluative. We screened citations by title and abstract for relevance to the assessment of acute pain in children. We attained the full-text publications and reviewed the reference lists of relevant studies.

This review was carried out in light of the surprising lack of research evidence and clinical practice that utilizes and integrates measurement of sensation together with assessment of affective and cognitive dimensions of children’s acute pain. As we argue throughout, that measurement or assessment of pain intensity alone is partial and captures only one part of the child’s broader pain experience. This review focuses on the internal pain experience and psychological aspects, rather than on functional outcomes (e.g., sleep disturbance, daily activities, school functioning, etc.) which are important to assess and consider but are outside the scope of the current review.

## 2. Pain: A Multidimensional Experience

The experience and impact of pain extends beyond the unpleasant sensory quality that defines the experience as painful. Accordingly, pain has been defined as an “unpleasant sensory and emotional experience associated with actual or potential tissue damage, or described in terms of such damage” [1], and although modifications have recently been suggested to the full definition, the multidimensional nature of pain remains integral [13]. Moreover, it has been well recognized since at least the early papers of Melzack and colleagues (e.g., [14]) that the pain experience encapsulates sensory-discriminative, affective, and cognitive-evaluative components. More recently, it has been proposed that the definition be expanded to acknowledge the importance of cognitive and social properties in individual experiences [15]. The sensory dimension encompasses perception of the location, intensity, and quality of pain. The affective dimension captures the unpleasantness and distress (which may encompass anxiety and fear) of pain and its implications [16]. The importance of the affective dimension of pain has been long recognized, notably with the ancient Greeks grouping pain with emotions rather than sensations [17]. The cognitive-evaluative component of pain involves the thoughts and appraisal of pain and may be guided by a motivation to terminate, reduce or escape from the painful event [18], being influenced by previous experiences, memory, expectations and knowledge [14]. The cognitive-evaluative dimension may include constructs such as pain-related catastrophizing and perceived threat, which drive both immediate and recalled perceptions of pain potentially fueling subsequent pain experience. Although cognitive-evaluative aspects of the pain experience have been more extensively researched in the chronic pain context, this may be problematic given that there may be marked differences in this pain dimension depending on whether the pain is acute or chronic [12]. Most notably, acute pain may be regarded as a protective mechanism, serving to promote recovery from injury. Older children, adolescents and adults who can appreciate such a view, are likely to hold very different interpretations of their experience. However, for young children, who lack the cognitive capacity to appreciate the distinction between chronic and acute pain, pain is pain, something to be avoided at all costs. The social features recognize that thoughts, expectations, appraisals, coping strategies, and emotions have early life origins in social experiences and are influenced by the social context.

**Figure 1:**
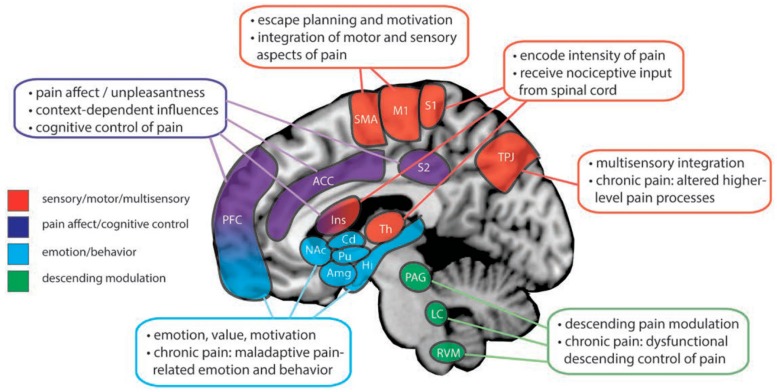
Summary of the main supraspinal regions and their roles in pain processing. Multiple cortical and subcortical structures are involved in various primary roles and aspects of the pain experience (as color-coded). Additional brain regions and networks not shown in the figure are involved in the pain experience. ACC = anterior cingulate cortex; Amg = amygdala; Cd = caudate; Hi = hippocampus; Ins = insular cortex; LC = locus coeruleus; M1 = primary motor cortex; NAc = nucleus accumbens; PAG periaqueductal gray; PFC = prefrontal cortex; Pu = putamen; RVM = rostral ventral medulla; S1 = primary somatosensory cortex; S2 = secondary somatosensory cortex; SMA = supplementary motor area; Th = thalamus; TPJ = temporal-parietal junction; © Wolter Kluwer (2018) Figure used with permission. Martucci KT, Mackey SC. Neuroimaging of pain. Human evidence and clinical relevance of central nervous system processes and modulation. Anesthesiology 2018; 128: 1241–1254. https://anesthesiology.pubs.asahq.org/article.aspx?articleid=2674194. The Creative Commons license does not apply to this content. Use of the material in any format is prohibited without written permission from the publisher, Wolters Kluwer Health, Inc. Please contact permissions@lww.com for further information.

Brain imaging in acute pain has provided conceptual support for multidimensional assessment of acute pain, through evidence of activation of different regions of the brain corresponding with different dimensions of the pain experience [19,20,21], as recently reviewed by Martucci and Mackey [22] and summarized in Figure 1. Complex neural mechanisms have been implicated for their impact on an individual’s pain experience, such as through multiple ascending spinal pathways to the brain with serial and parallel processing in the brain [18].

Encoding of the intensity of painful stimuli is thought to be associated with the primary somatosensory cortex and posterior insular cortex [23,24]. The affective dimension of pain is related to perceptive and context-dependent pain processes within the brain [22] and may involve regions such as the secondary somatosensory cortex and anterior insular cortex [25]. Increased blood flow in the posterior parietal and prefrontal cortices is thought to reflect attentional and memory networks activated by noxious stimulation [20], supporting further neurobiological underpinnings of the cognitive-evaluative dimension of pain.

Signals from the peripheral nervous system undergo complex modulation by affective and cognitive-evaluative processes when they enter the central nervous system. Converging evidence suggests that the prefrontal cortex (e.g., anterior cingulate cortex, ventromedial prefrontal cortex, dorsolateral prefrontal cortex) is involved in the cognitive modulation of pain [22,26,27,28,29,30]. Motor and supplementary motor cortices may be related to the motivational aspects of the pain experience [31]. The amygdala has been identified as having an important role in the affective dimension of the pain experience [30,32] and, together with the hippocampus, contributes to the memory processing of pain [33]. Intensity discrimination, motor response, and motivational aspects of pain may be influenced by the activation of sub-cortical structures within the basal ganglia [22,34]. As depicted in Figure 1, the brainstem, midbrain, and medullary regions, such as the midbrain periaqueductal gray, locus coeruleus, and rostral ventral medulla, are involved in the descending modulation of pain [22,35,36].

It is widely recognized that attentional modulation, involving the descending pain modulatory systems, may impact pain perception [37,38]. There is also evidence that attentional modulation may engage the ventrolateral prefrontal cortex, leading to a change in the emotional significance of the pain [38]. Furthermore, pain catastrophizing results in activation of brain regions that are not generally associated with sensory-discriminative aspects of pain, such as the primary or secondary somatosensory cortex [39]. As pain persists to chronicity, there is generally a shift from pain regions engaged in processing the sensory component of pain towards regions that encode emotional and motivational subjective states [40]. The human brain has been described as a social brain [41], thereby implicating social influences in cognitive and affective processing.

Although neuroimaging techniques have greatly enhanced the understanding of the multidimensional nature of the pain experience, most studies have been with adults, and in acute procedural pain more than postoperative or posttraumatic clinical acute pain. More neuroimaging studies investigating the various dimensions of the pain experience are needed with children considering their developing brains.

The multidimensional nature of the acute pain experience is further evidenced by the fact that sensory and affective pain qualities have been found to be differentially expressed in facial responses [16]. Specifically, facial movements around the eyes were found to be more associated with elevated reports of sensory aspects of the pain, whereas movements of the eyebrows and upper lip were more associated with the affective pain dimension [16]. Kunz et al. [16] concluded that the facial expression of pain is a multidimensional response system that differentially encodes sensory and affective dimensions of the pain experience.

Theoretical, experimental and clinical streams of evidence seem to be converging on the distinctiveness of the various dimensions of pain. Whilst sensory, affective and cognitive-evaluative components are widely regarded as central, yet distinct aspects of the pain experience [42], pain measurements and assessments in the clinical pediatric acute pain context currently tend to focus predominantly, if not exclusively, on the sensory domain. As yet, social features of pain remain relatively unexplored [43].

## 3. Concordance and Discordance between Various Dimensions of the Acute Pain Experience

Although children’s ratings of pain intensity often correlate with pain affect, the two dimensions, and their relations with pain outcomes, have also been found to diverge. Regarding immediate pain, self-report ratings of pain intensity and affective measures, such as unpleasantness, are often moderately to highly correlated [44,45,46,47]. However, this finding is not universal and some studies have reported more modest correlations [48]. This may, in part, be related to different scales measuring different features of the complex pain response, or it may be related to contextual differences. In some situations, children may find the affective aspects of pain to be more troubling than sensory aspects [49]. One pediatric study that was carried out in the context of routine needle insertion into a subcutaneously implanted intravenous port, found that when topical anesthesia was used, needle-related fear was significantly higher than needle-related pain intensity [50].

The distinct nature of the various pain dimensions, and the ability of children to discriminate between these, was apparent in a study examining children’s multidimensional ratings of a needle-pain experience [46]. Children’s pain intensity ratings using a facial scale were more highly correlated with a visual analogue scale for pain intensity than a visual analogue scale for unpleasantness [46]. Likewise, scores using the Facial Affective Scale were more highly correlated with a visual analogue scale for unpleasantness than a visual analogue scale for pain intensity [46]. Similarly, discriminant validity has been established between measures of 5- to 10-year-old children’s pain-related fear, using the Children Fear Scale and measures of pain intensity, using the Faces Pain Scale–Revised, in the venipuncture context [51]. Moreover, in the post-surgical context, self-reported scores obtained from 5- to 15-years-old using a facial pain intensity scale have been found to be correlated more highly with an analogue measure of pain intensity than an analogue measure of unpleasantness [47,52].

Taken together, these results provide evidence for both concurrent and discriminant validity of measures assessing various sensory and affective pain constructs. Hence, not only do there seem to be distinct sensory and affective pain dimensions, but children as young as 5 years have demonstrated their ability to discriminate between these dimensions.

Arguably, pain catastrophizing is the most well researched cognitive-evaluative pain construct in the pediatric acute pain context; albeit much of the research has been carried out in the experimental [52] or surgical pain [53] contexts. It has been suggested that children who are higher in pain catastrophizing are more communicative about their pain, and therefore, are more likely to report feelings of distress and or high pain intensity [54]. As well, it is argued that these individual differences in cognitive coping and pain communication have origins in early life social experiences, attesting to the importance of the social environment [55,56,57,58]. Correlations between children’s pain-related catastrophizing and measures of pain intensity or unpleasantness have ranged widely and are arguably dependent on contextual and participant factors [52,54].

When considering the relationships between the various pain dimensions, it is important to recognize that different pain dimensions may be more or less salient to the individual at different stages of the acute pain experience. In the anticipation phase, prior to exposure to the nociceptive stimulus, the affective and cognitive-evaluative dimensions are likely to dominate, whereas the sensory dimension is likely to be more salient during the encounter and recovery phases. However, the human, as an integrated organism, is constantly adapting to the environment, and therefore experiencing and responding to pain in an integrated fashion. Hence, the various phases of the pain experience do not occur independently of one another, and a child’s experience during the anticipatory phase may have a strong bearing on how they respond in the encounter and recovery phases. For example, children who anticipate higher levels of pain intensity, subsequently report experiencing greater pain intensity [59,60]. Children’s anticipatory distress has also been found to be positively associated with greater subsequent pain intensity experienced during a medical procedure [61], an acute experimental/laboratory pain experience [62], and in the post-operative context [63]. A similar relationship has been found when considering the caregiver’s perspective. During lumbar punctures and/or bone marrow aspirations, higher anticipatory caregiver distress was linked to caregivers subsequently reporting higher proxy ratings of the child’s pain intensity [64].

Beyond immediate/experienced pain, research on children’s memory for pain—a key cognitive-evaluative dimension of the pain experience—reveals important differences between recall of pain intensity versus pain affect. This provides support for the distinctiveness and relative clinical significance of children’s recall of the sensory versus affective dimensions of pain. Indeed, low correlations between these dimensions of recalled pain are often observed (for a review see [65]). Moreover, in a recent investigation of 5- to 7-year-old children undergoing tonsillectomies [66], low correlations were found between current and recalled pain intensity ratings, as well as relationships between recalled pain intensity and recalled pain-related fear. In a study of venipuncture pain, Lander and colleagues [67] found that children’s recall of pain was more accurate (i.e., recalled pain corresponded to current pain ratings) for the affective, rather than sensory, aspects of the pain experience. This provides further support for the distinctiveness of sensory versus affective aspects of pain memory, as initially argued by Peter Ornstein [68]. It is also in keeping with existing literature that has repeatedly shown differential relationships between key variables and children’s memory for affective versus sensory aspects of pain [65]. Indeed, research has found significant associations between individual factors, such as anxiety or pain catastrophizing, and children’s recall of pain-related fear, but not recall of pain intensity [66,69]. That is, higher anxiety and catastrophic thinking about pain have been found to lead to negative biases in recall (i.e., recalled pain is higher than current pain ratings) for the affective, but not the sensory, aspects of pain experiences.

Finally, the complexity regarding the inter-correlations between the various dimensions of pain is amplified when considering the developmental changes throughout childhood and adolescence. The way in which children respond to various aspects of pain experiences evolves with development. Children aged 5 to 7 years have been found to be more likely to characterize their pain in sensory and affective terms, whereas older children are likely to refer to more cognitive-evaluative aspects of the pain experience [70]. At least in the needle pain context, age has been found in one study to be more positively associated with pain intensity ratings than unpleasantness ratings [45]. In contrast, gender differences have been reported as more likely to emerge when reporting on unpleasantness rather than sensory, dimensions of the needle pain experience, with girls giving higher unpleasantness ratings than boys [45].

It has been suggested that children aged under 8 years may experience more difficulty discriminating between pain intensity and pain-related unpleasantness [45]. It is possible that in some cases the self-reported pain intensity ratings of young children may be more than a rating of sensory intensity, but rather an integration of the various pain dimensions. However, even if young children understand the different constructs, rating multiple dimensions of a pain experience may require a degree of cognitive flexibility necessary for switching between different constructs that exceed the abilities of preschoolers. Nevertheless, not all studies have found higher correlations between intensity and unpleasantness in young children (cf [47]), suggesting that even young children may, in some circumstances, be able to discriminate between these constructs.

Although considerable attention has been devoted to the developmental changes in the way children self-report pain intensity throughout childhood (e.g., [71]), much less is known about how children experience and self-report affective and evaluative pain dimensions throughout childhood. This is most notably manifested through a lack of age-appropriate tools to assess cognitive-evaluative or social aspects of the acute pain experience in young children [72].

Taken together, these findings collectively point to the complex and dynamic interactions between the various pain dimensions, highlighting the value of age-appropriate multidimensional assessment within a temporal context [73].

## 4. Pediatric Multidimensional Acute Pain Measurement in the Clinical Context

Numerous modalities of acute pain measurement may be used in clinical practice, including behavioral (observational), physiological and self-report measures. Physiological indicators may be useful in the absence of other sources of information, however, they may be confounded with various psychological and medical factors. With the exception of detailed and time-consuming facial coding [16,74], there is little evidence to support the ability of behavioral (observational) pain measures to discriminate between the various dimensions of the pain experience [75,76,77]. Self-reported pain assessment is the only assessment modality with the potential to offer a nuanced, multidimensional assessment, limited only by the cognitive and reporting ability of the individual.

One of the primary purposes of pain assessment in the acute clinical context is to help guide therapeutic interventions. Arguably, this is likely to be better achieved if pain assessment is multi-dimensional rather than unidimensional, potentially enabling better identification of individuals at greater risk of poorer long-term outcomes and pain chronicity, and more tailored therapeutic interventions. These potential benefits will be considered in more detail.

### 4.1. Acute Pain Dimensions Associated with Transition to Chronicity

Over the past decade, increasing attention has been devoted to the transition from pediatric acute pain to more chronic pain disorders, with numerous studies investigating the ability of various sensory, affective, and cognitive-evaluative factors in the pediatric acute pain (principally surgery) context to predict the development of persistent or chronic pain disorders [78,79,80,81,82,83]. A meta-analysis investigating predictors of pediatric chronic post-surgical pain found that pre-surgical measures of pain intensity, child anxiety, and child pain-coping efficacy were all predictors of chronic post-surgical pain [82]. Several studies have also revealed the powerful roles of parental and child anxiety-related constructs, both at baseline and in the immediate acute recovery phases, in predicting the development of pediatric chronic post-surgical pain [79,80,84]. Additionally, anxiety-related factors have been shown to be robust risk factors for the development of negatively biased pain memories [85], which have been demonstrated to underlie the transition from acute to chronic post-surgical pain [81]. These affective and evaluative predictors of chronicity, often reflecting the child’s life experiences in social contexts, are likely to operate within the broader context of genetic vulnerability to chronic pain [86], as well as an associated imbalance between enhanced ascending nociceptive inputs, such as central sensitization, and inadequate inhibitory descending pathways [87].

Routine assessment of children’s multidimensional pain experience (before, during and following the inciting painful event), particularly the affective and cognitive-evaluative aspects, may provide a clearer understanding of factors impacting acute pain trajectory, the rate of resolution, and the transition to chronicity. This will enable the identification of children at greatest risk of poor long-term pain outcomes and may guide the development of targeted early interventions.

In addition to genetic [86] and physiological [87] accounts for the development of pain chronicity, various psychological theories have been proposed to account for why some individuals subsequently develop chronic pain whereas others do not. These psychological theories hold particular value given that psychosocial factors tend to be more modifiable than biological factors, providing greater potential for early intervention. The seminal models proposed in the adult literature have been the Fear Avoidance Model [88] and Threat Interpretation Model [89], amongst others [90]. Of note is that these theories all highlight the critical, and often primary, importance of affective or evaluative pain dimensions in contributing to pain chronicity. For example, the Fear Avoidance Model holds that upon confrontation of an acutely painful stimulus, individuals who engage in more catastrophic thinking about pain will be more likely to experience greater pain-related fear and anxiety, which serves to further fuel avoidance behaviors, depression, and disability. As a result of these interacting affective processes, individuals experience chronification of their pain. This occurs in the context of developmental processes and familial socialization which may augment the cognitive and affective dimensions of pain and are an important target for psychological and social interventions. When considering the Threat Interpretation Model and the role of attentional biases, it is important to acknowledge key differences between the acute and chronic pain contexts. Identifying cues as threatening in the acute pain context may be useful and serve to avoid further damage and to promote healing. However, little data are available on whether individuals who are highly vigilant to potential threats in the acute context are more likely to remain highly vigilant after the physical damage has healed, thereby leading to poorer functioning and higher risk of pain chronicity.

More recently, fear avoidance models have been extended to pediatric populations [91,92]. While many of the same intrapersonal affective factors are posited to play similar contributory roles in pain chronification, the critical role of interpersonal, and particularly caregiver, affective factors are underscored. Specifically, the models posit that when a caregiver is confronted with their child in pain, this can be perceived as threatening. If this engages an aversive affective response, self-oriented distress, and increased catastrophic thinking and fear, this can lead to solicitous or protective responses that further ignite children’s own catastrophic appraisals, fear, anxiety, and ultimately avoidance and disability. Given the role of observational learning in the development of pain beliefs and behaviors [57], these cognitive-affective and behavioral responses of caregivers interact with those of children and are considered critically important in the self-perpetuating cycle of fear-avoidance thought to lead to the development and maintenance of chronic pain in youth.

Hence, there is both an empirical and theoretical basis for why multidimensional acute pain assessments targeting sensory, affective and cognitive-evaluative domains may be valuable in identifying which pediatric patients are likely to transition to more chronic pain problems. The relationships are likely complex, interacting with other behavioral, social and developmental factors, as well as with an array of neurobiological pathways, to shape long-term pain outcomes, such as disability [93]. As a point of perspective, it is important also to highlight the neurobiological changes that underlie the progression from acute to chronic pain [94], which in turn are influenced by the cognitive-evaluative and emotional experiences.

### 4.2. Multidimensional Pain Assessment and Therapeutic Decision-Making

Multidimensional pain assessment may provide an opportunity for more tailored therapeutic interventions. Even though some treatment algorithms and clinical guidelines are based on pain intensity scores (e.g., World Health Organization Analgesic Ladder), pain intensity scores alone are a poor guide for clinical intervention, failing to take into account other relevant information [10,95]. It is important, therefore, to consider how a more comprehensive pain assessment may be used to inform therapeutic response.

Little is known about how clinicians currently respond to children’s self-reported pain responses, especially when these extend beyond pain intensity scores. If clinicians do ask patients about pain-related affective or evaluative dimensions, it is currently more art than science as to how they then integrate and respond to this information alongside other physiological and behavioral pain indicators and management protocols. It has been suggested that clinician acute pain treatment decisions are typically based on the presumed mechanism of the pain, previous pain medication use, and clinician experience [3], and there has been little, if any, attention devoted to how best to integrate functional measures, behavioral observations, and children’s self-report with such factors. One study found that clinician estimations of a child’s procedural pain intensity were influenced by the child’s diagnosis, the child’s pain behaviors and the clinician’s own distress in the anticipatory phase, with higher clinician distress predictive of greater subsequent pain intensity ratings [64]. Although not directly assessed, it was speculated by Caes et al. [64], that these factors may, in turn, impact on clinician pain management decisions. Notably, a study within adult pain context identified a range of psychosocial influences on clinician appraisal of pain [96].

The potential exists for elevated responses on specific pain dimensions to flag the need for clinicians to consider particular therapeutic interventions. Alternatively, different multidimensional pain profiles may signal the need for certain interventions or treatment packages. These issues are considered in more detail in the section on Future Directions (Section 7).

## 5. Pediatric Acute Pain Assessment for Clinical Trials

Clinical trials aim to determine whether interventions provide a valid, measurable and clinically meaningful improvement. Considerable work has been carried out to identify the clinical significance of pain intensity, but not affect, change scores. For example, one study found that a two-point change on the Faces Pain Scale–Revised or a one-point difference on the Coloured Analogue Scale (scored out of 10) was considered by children as reflecting “a little less” pain, and a three-point reduction on both scales reflected “much less” pain [97]. However, the minimum clinically important difference is likely to be influenced by the baseline pain level, whereby a change of two points from 2/10 to 0/10 may not be experienced by the patient in the same way as a change from 9/10 to 7/10 [98,99,100]. Other researchers have applied more stringent criteria of 50% reduction in pain intensity scores as corresponding with patient impressions of improvement. Again, this has not been examined from a multidimensional perspective.

Although different methods have been used to calculate whether change in pain intensity scores reflect clinically meaningful improvement, Sloman et al., [100] concluded, based on their results with adults, that a pre- to post-analgesia shift in numeric pain intensity scores was not a good indicator of the clinical relevance of the shift from the patient’s perspective, as baseline pain intensity needs to be accounted for [100]. However, one may speculate that the lack of clinical relevance may also be underpinned by the reliance on only a unidimensional, sensory-based pain measure, given that affective and cognitive-evaluative aspects also likely impacted on patient perspectives of improvement. There is increasing recognition that clinical trials need to adopt a more holistic assessment of clinical significance, utilizing multiple measures of key pain outcomes [101]. The clinical significance of an individual’s painful experience is unlikely to be well summarized in terms of only the intensity dimension.

Widely-cited recommendations from the Pediatric Initiative on Methods, Measurement, and Pain Assessment in Clinical Trials (PedIMMPACT) have identified a range of core outcome domains and measures that should be considered when evaluating outcomes in clinical trials for acute pain in children and adolescents [101]. When conducting clinical trials in the pediatric acute pain context, it is recognized that measures of pain intensity should be supplemented with measures of global judgment of treatment satisfaction, symptoms and adverse events, physical recovery, emotional response, and economic factors. Such guidelines have influenced funding bodies, which increasingly require more than just a unidimensional indicator of change as evidence of effect. Although clinical trials may be moving closer to achieving multidimensional pain assessment, clinical practice remains a long way from these standards.

## 6. Possible Reasons for Why Acute Pain Assessment in Children Is Often Unidimensional

Whilst few would question the multidimensional nature of pain, nevertheless, unidimensional assessments focusing on pain intensity, typically supplemented with clinical observations, are common in clinical pediatric acute pain contexts. A range of historical and pragmatic reasons may help explain why unidimensional pediatric acute pain assessments are so prevalent (see Table 1). We provide the apparent reasons for the focus on unidimensional assessment along with a rationale and recommendations for broader multidimensional assessment.

*Reason 1.* A typical ward round by an Acute Pain Team in a busy pediatric hospital or ward only allows for relatively brief interactions with patients and their families. In this context, unidimensional acute pain assessment tools may be perceived as offering a more pragmatic, relatively objective, time-sensitive and user-friendly means of informing pain relief decisions. This is reflected in the World Health Organization Analgesic Ladder which relies on pain severity to guide analgesic choices. There is a need for clinically acceptable, bedside suitable, pediatric multidimensional acute pain assessment methods which better reflect biological, psychological, emotional, social and cultural realities, and accommodate different developmental, educational, cognitive and social contexts.

*Reason 2.* Some clinicians may hold the misguided view that pain intensity is necessarily the most bothersome and troubling dimension of the acute pain experience. In a study that utilized a multidimensional acute pain assessment protocol in an adult post-operative population, one in ten patients reported “unacceptable” pain, yet a low pain intensity score [102]. This suggests that pain intensity is not representative of an individual’s whole pain experience and that unidimensional acute pain assessment risks undertreating patients. It has been suggested that gauging the perceived impact that pain has on an individual and their functional capacity, has more significant treatment implications than a measure of pain intensity alone [102]. Multidimensional acute pain assessment, with an inter-disciplinary team approach, will better inform the multi-objective optimization of acute pain management [103].

*Reason 3.* Some hold the misperception that an individual’s cognitions and affect associated with a painful experience predominantly occur in reaction to the sensory dimension [93]. A logical extension of this misperception would therefore be that appropriate assessment and management of the sensory pain dimension would be sufficient. However, assigning the cognitive and affective dimensions, secondary status stands in contrast to considerable empirical work which highlights complex bidirectional influences between the sensory, affective and cognitive-evaluative pain dimensions (for reviews see: [18,93]). Although various psychosocial factors may influence how individuals report their pain, this does not diminish the importance of affective and cognitive aspects of the actual pain experience.

*Reason 4.* Healthcare professionals might feel better equipped to manage the sensory dimension of pain, in contrast to the affective or cognitive-evaluative dimensions, and may therefore avoid assessing the other dimensions. This may be because medical education and training on the effective management of acute pain focuses predominantly on pharmacological interventions for reducing pain intensity [104]. It is known that suboptimal knowledge of the pharmacodynamics and pharmacokinetics of pain medications leads to under-treatment of pain intensity [105]; therefore, it is likely that deficient knowledge of management options for other dimensions of pain similarly leads to sub-optimal pain management. If the administration of multidimensional acute pain assessment tools is to translate into improved pain outcomes and patient satisfaction, continuing professional development and healthcare education curriculums should include multimodal integrative management options, such as psychoeducation, distraction and other attentional techniques, mindfulness training, cognitive-behavioral interventions, and hypnotic techniques [106,107]. This may need to be accompanied by a shift in targeting analgesic therapy at the sensory dimension of pain intensity, to the contributing and related dimensions of acute pain for each patient [108].

*Reason 5.* The predominant use of mechanistic, unidimensional acute pain assessment methods may be an unintended legacy of the 1990′s ‘Pain: the 5^th^ Vital Sign’ campaign. This campaign drew attention to the importance of acute pain assessment by mandating routine screening of pain intensity [109]. Whilst the campaign contributed to the current ubiquity of acute pain assessment, these assessments usually comprised a single numerical rating of pain intensity to record in the patient’s medical chart. Routine acute pain assessment often does not extend beyond quantifying pain intensity, and a purely perceptual model of pain is at best incomplete. What began as an innovative initiative to measure acute pain now leaves many clinicians often failing to fully appreciate the biopsychosocial complexity of the acute pain experience, sometimes devising entire pain management plans based on a mere numerical value for pain intensity [110], consequently resulting in limited improvements in patient outcomes [9,111]. The focus now needs to be on a conceptual and cultural shift from unidimensional assessment of acute pain to a more comprehensive, multidimensional assessment of acute pain.

*Reason 6.* While some single item scales exist to assess children’s pain-related affect in the acute pain context (e.g., Children’s Fear Scale to assess pain-related fear [51], Facial Affective Scale [112], there is an overall dearth of age-appropriate, valid and reliable tools to assess non-sensory pain dimensions in children. Moreover, there are very few tools that are designed to assess multiple dimensions of a child’s pain experience and are useable in the clinical context (e.g., Adolescent Pediatric Pain Tool [113,114]; Children’s Anxiety and Pain Scale [115]). Most existing multidimensional pain assessment tools are either designed for chronic pain and their length makes them impractical for assessing acute pain, or designed for acute pain in a research context and are unsuitable for informing clinical decision-making in a clinical care setting [9]. Evaluative aspects of the pain experience (e.g., pain-related catastrophizing, perceived injustice, perceived threat) are most commonly assessed using questionnaires. Such measures are well suited for use in a chronic pain context, or perhaps in clinical trials, but are generally more time-consuming to complete than is feasible on a busy acute pain round. New paradigms for assessing affective and evaluative dimensions of pain need to be explored in the acute pain context. A notable existing measure is the Clinically Aligned Pain Assessment (CAPA) Measure, which is a conversation guide that assesses comfort, changes in pain, pain control, functioning, and sleep [116] and has been found to have preliminary pragmatic validity and acceptability. Although this measure is a significant advance to unidimensional, sensory measures, it does not assess the affective or cognitive-evaluative pain dimensions. Another existing multidimensional pediatric pain assessment measure is the Adolescent Pediatric Pain Tool (APPT; [113,117]), which not only elicits information about pain sites and pain intensity, but also requires respondents to identify which sensory, affective and evaluative word descriptors best describe their pain experience. The APPT has been developed for use with children and adolescents aged 8–17 years, and given the complex and nuanced use of vocabulary is not suitable for younger children or individuals with poor language ability. Given the relative paucity of evidence that existing pain assessment tools in general are an effective or efficient aid in diagnosing or managing pain [118], there is a need for novel tools assessing the affective and evaluative dimensions of pain that are validated with young children and which maximize time cost-benefit.

Although considerable work has been carried out documenting the cognitive pre-requisites associated with the ability to self-report pain intensity [71], much less is known about the cognitive requirements associated with the ability to self-report other aspects of the pain experience. Moreover, less attention has been devoted to the development of assessment tools to assess other aspects of the pain experience, such as cognitive and evaluative dimensions, particularly in young children. Although age-appropriate self-report scales have been found to provide meaningful information about a child’s pain intensity from as early as the age of 4 years, right through into adulthood [119,120], much less is known about the ability of young children to provide meaningful self-reports of other dimensions of the pain experience.

*Reasons 7.* There is recognition of the importance of affective dimensions of pain, but a lack of clarity regarding what is meant by affect. Pain affect is not a unitary construct, but rather can mean a range of different experiences, including unpleasantness, distress, fear, anxiety, sadness or disgust. Not only have different affective constructs been found to be associated with the activation of different brain regions [121], but importantly, these constructs do not necessarily relate to pain reactivity and intensity in the same way. For example, an experimental study with adults found that induced fear decreased pain reactivity, whereas induced anxiety increased pain reactivity [122]. To add to the confusion, it is not uncommon for researchers and clinicians to use the terms loosely and without consideration of the wealth of literature defining the constructs.

*Reason 8:* Health professionals who deem the available acute pain assessment tools as clinically ineffectual, conceptually incomplete or administratively complex, are likely to avoid using these tools and may filter the patient’s self-reported pain through learned heuristics. Whilst this work-around behavior is likely performed in the interests of management simplicity and patient safety, it generally undermines an appreciation of the multidimensional nature of acute pain [123]. Furthermore, there is evidence of considerable variability in the ability and motivation of humans when assessing pain in others [118], a problem that is amplified when standardized tools are not used.

*Reason 9:* Some health professionals may hold the view that children lack the cognitive maturity to be able to provide meaningful self-reported information on more than a single dimension of the pain experience. However, this is likely to reflect an underestimation of the capabilities of children. Notably, a study conducted with preschool children, not experiencing current pain, found that many 4-year-olds were able to switch between reporting on different (non-pain-related) constructs [124]. Further research is needed in the acute pain context given that attentional flexibility between various dimensions may be more challenging in stressful contexts [125]. Drawing from the cognitive-developmental literature, switching between a more salient dimension to a less salient dimension may be more effortful than if the dimensions were similar in salience [126]. It is not known how this may impact on a child’s ability to self-report multiple pain dimensions if one dimension, such as pain-related distress, was particularly salient for the child. Although more cognitive-developmental research is warranted in the context of children’s multidimensional pain reports, it is likely that children’s ability to provide meaningful multidimensional self-reports of their pain experience is more limited by the availability of age-appropriate, validated assessment tools, rather than by a lack of cognitive capacity per se.

## 7. Future Directions

If the potential benefits of multidimensional acute pain assessment are to be actualized in the clinical pediatric acute pain context, further work is needed in the development of age-appropriate affective and evaluative self-report assessment tools [9], research into the potential clinical application and value of multidimensional assessment [1], education of health professionals on the multidimensional nature of the acute pain experience and possible assessment methods [14], and expansion of multidimensional assessment to include the child’s social context [15]. These areas will be discussed in more detail.

### 7.1. Development of Age-Appropriate Self-Report Affective and Evaluative Pain Assessment Tools

Just as an artist needs sharp pencils to depict the detail of their image, so too a health professional needs valid and reliable assessment tools which possess the precision to obtain meaningful information from patients about different dimensions of their pain experience. Although much research has been devoted to the development of age-appropriate tools for assessing children’s self-reported pain intensity (e.g., [7,127]), more work is needed to develop age-appropriate tools to assess pain-related affective, cognitive-evaluative and social dimensions in a clinical acute pain context.

Research is needed into whether existing analogue scales that have been validated for the assessment of pain intensity may be used to assess aspects of pain-related affective or cognitive-evaluative dimensions, with modified verbal anchors, as exemplified by Vaegter et al. (2017) in the adult context [128]. The extent to which children, especially young children, may select the same response options without distinguishing between the different anchors when the tool is used to assess different constructs, needs to be evaluated. Consideration should also be given to whether switching between different pain dimensions is cognitively challenging for young children and whether one construct, either the first construct assessed, or perhaps the construct that is most salient to the child, may influence their other ratings. It may be beneficial if pain assessment is initiated by eliciting the child’s perspective about whether the pain intensity or the pain-related affect is more salient for them.

Relatively little is currently known about the ability of children, especially young children, to report on cognitive-evaluative aspects of pain. This dimension has mostly been assessed using questionnaires, thereby being limited by the reading age of the child. Questionnaire format enables more detailed and nuanced questions, however, it takes considerable time, and therefore may be impractical for use in the acute pain clinical context. Given the limited research in this area, some questionnaires assessing cognitive-evaluative aspects of the adult pain experience, such as pain catastrophizing and perceived injustice, have been adapted for use with children and adolescents [129]. Although adaptations may ensure that age-appropriate language is used, questionnaires that have been developed with adults may use concepts that lack relevance for children, who think differently to adults [130]. Not only is there a need to develop new child-specific questionnaires of cognitive-evaluative pain constructs, but there is a need for new assessment paradigms for assessing cognitive-evaluative pain constructs in acute clinical contexts, particularly with young children.

### 7.2. Future Research into the Clinical Application and Value of Multidimensional Pediatric Acute Pain Assessment

Arguably, the primary purpose of acute pain assessment is to optimize therapeutic response; however, evidence-based guidance is lacking on how clinicians should respond to multidimensional assessment reports. The concept of tailoring interventions and clinical decision-making based on a multidimensional assessment has considerable appeal. Along these lines, it has been suggested that analgesic drugs are generally appropriate for pain of a high intensity, while psychosocial interventions may be more appropriate for pain with a high affective component [131]. However, scores on individual pain dimensions should not be considered in isolation. It would be inappropriate to prescribe opioid analgesics solely on pain intensity scores, without considering other relevant factors [95]. Nor do pain interventions work exclusively on one pain dimension. Notably, psychological interventions that address emotions simultaneously also modulate perceived pain intensity [132]. Hence, when considering the potential for tailoring interventions to multidimensional pain scores, it may be fruitful to establish whether individuals with certain pain profiles are most likely to benefit from certain interventions or intervention packages. Although most pain interventions have the potential to simultaneously impact on multiple pain dimensions, some interventions have been developed to primarily target particular mechanisms and therefore seem to be more closely aligned with certain pain dimensions. For example, opioid receptor agonists block messages of pain, thereby reducing the perceived intensity of the pain experience. Mindfulness interventions for pain center around the ability to attend to the pain experience without judgement or cognitive evaluation [133], thereby aligning most closely with the cognitive-evaluative dimension of pain. Memory-reframing interventions also focus on modifying any inaccuracies in the child’s recall and cognitive evaluations about a painful experience [134]. However, given that each of the interventions is known to concurrently impact on numerous sensory, affective and cognitive-evaluative aspects of pain, it may be beneficial to consider whether clinical decisions and intervention packages may be best tailored to multidimensional profiles rather than individual construct scores. Further research in this area is warranted.

With considerable research over the past decade devoted to identifying the long-term predictive value of affective and cognitive-evaluative dimensions of the pain experience [78,80,81,82,84], attention needs to be directed to translating this knowledge into clinical practice. Targeted early interventions may be developed for children experiencing acute surgical or other pains, who are identified, based on their multidimensional pain assessment, as being at risk of pain chronicity. To date, there has been a dearth of research into the potential of early interventions [135].

Further research is needed to establish how the concept of suffering aligns itself with the multidimensional nature of pain. Suffering has generally been defined in cognitive terms, such as “the perception of serious threat or damage to the self” [136]. Consideration is needed to establish whether suffering is primarily dependent on an individual’s cognitive evaluation of their pain experience or an individual’s threshold of acceptability for the various pain dimensions. It has been suggested that suffering emerges when a discrepancy occurs between one’s expectations of one’s self and one’s actual experience of self [136].

The current paper has focused on multidimensional pain self-reporting. However, self-reported information about an individual’s clinical pain experience should be considered within a broader social and developmental context, and considered alongside behavioral observations, physiological measures (where available), an understanding of the pain mechanism and pathophysiology, and the patient’s history. There is currently little to guide clinicians about how to integrate these various sources of information. It needs to be acknowledged that although behavioral and physiological measures of pain are informative, they do not distinguish between the various pain dimensions in the way that self-reported assessment can.

### 7.3. Education of Health Professionals Regarding Multidimensional Pain Assessment

In light of the previous discussion of possible barriers to the multidimensional assessment of children’s acute pain, the importance of well-informed pain education for health professionals should not be understated. It would be valuable if education programs highlighted the multidimensional nature of the pain experience, acknowledging the importance of the affective and evaluative pain domains, rather than assuming these to be secondary consequences of pain intensity. As more affective and evaluative pain assessment tools are developed for children, it is important that health professionals are trained in the use of such tools. Moreover, educators and policymakers should guide health professionals in how to record multidimensional self-reported pain scores and how these can be assimilated with other sources of information gathered.

### 7.4. Expansion of Multidimensional Pain Assessment to Include the Child’s Social Context

An individual’s social context is critically important in shaping their pain experience. Indeed, the social dimension has been proposed to be included in the very definition of pain [15]. Particularly when considering the pediatric period—across infancy, childhood, and adolescence—caregivers exert a powerful role on children’s experience of pain. Theoretical and conceptual models of acute and chronic pediatric pain posit that the cognitive-affective experience of caregivers when observing their child in pain can fundamentally alter all dimensions of the child’s pain experience [91,92,137]. Perhaps the most compelling demonstration of this has been in the post-surgical pain context. Parental catastrophizing about their child’s pain prior to surgery has been shown to predict persistent post-surgical pain trajectories [84]. Moreover, parental catastrophizing has been found to be a more important predictor of negative biases in children’s pain memories than the child’s own cognitive-affective state [69]. Whilst this has been demonstrated in the adolescent period, the influence of parental cognitions and affect is thought to be even more influential during earlier developmental periods [69,91]. A recent examination of 5- to 7-year-old children undergoing surgery demonstrated that parental, but not child, anxiety predicted children’s later recall for the affective dimension of pain [66]. Importantly, these effects are not trivial or short lasting. A parent’s behavior during their infant’s vaccine injections during the first year of life was found to predict the degree of anticipatory distress that the same child experienced during vaccine injections during the preschool period [138]. As such, there are strong grounds for suggesting that multidimensional pain assessment in children be expanded to capture affective and cognitive-evaluative dimensions of their caregiver’s experience.

### 7.5. Limitations

It should be acknowledged that there has been limited empirical work in this field, largely attributable to a lack of age-appropriate, validated multidimensional self-report assessment tools. Consequently, this narrative review has drawn heavily on conceptual and theoretical considerations, paving the way for further research.

## 8. Conclusions

Despite pain being widely acknowledged to be a multidimensional experience, sensory-focused, unidimensional pain assessments are still common in the pediatric acute pain context. Multidimensional acute pain assessments that tap into sensory, affective and evaluative dimensions offer a better fit with theoretical conceptualizations of the pain experience. The ability to use multidimensional acute pain assessments to identify individuals who are at risk of poorer long-term outcomes and pain chronicity would enable targeted early interventions. Given the disproportionate emphasis devoted to the sensory component of the pain experience in children, more research is needed into the development of age-appropriate self-report tools to assess affective and evaluative dimensions of the pain experience. New assessment paradigms need to be developed to acquire meaningful self-report from younger children in these domains. Pediatric acute pain assessments that utilize age-appropriate and validated measures of sensory, affective and evaluative pain dimensions have considerable potential value, but must also be accompanied by clinical observation, appropriate history taking and a broader consideration of the social context.

## Figures and Tables

**Table 1:** Possible reasons why acute pain assessment in children is usually unidimensional.

Reason 1	Time constraints of acute pain ward rounds.
Reason 2	Misperception that the sensory dimension of pain is the most bothersome aspect of pain.
Reason 3	Misperception that the sensory dimension of pain is primary and the affective and cognitive dimensions occurs as a reaction to the sensory experience.
Reason 4	Healthcare professionals may feel better equipped to manage the sensory dimension of pain and may avoid assessing other dimensions.
Reason 5	Unintended legacy of the ‘5^th^ Vital Sign campaign’, whereby nurses who were trained and mandated to include a pain intensity score with their routine observations may feel that this is sufficient.
Reason 6	Inadequate availability of validated tools to assess other dimensions of pain in children, especially young children.
Reason 7	Lack of clarity of what is meant by affect (e.g., unpleasantness, distress, fear, disgust etc.), consequently making it difficult to assess the affective dimensions of pain.
Reason 8	Healthcare professionals may perceive existing tools to assess pain as clinically ineffectual, conceptually incomplete or administratively too complex.
Reason 9	Belief by health professionals that children lack the cognitive maturity to self-report more than a single dimension of the pain experience.
